# Persistent Circulation of Enterohemorrhagic *Escherichia coli* (EHEC) O157:H7 in Cattle Farms: Characterization of Enterohemorrhagic *Escherichia coli* O157:H7 Strains and Fecal Microbial Communities of Bovine Shedders and Non-shedders

**DOI:** 10.3389/fvets.2022.852475

**Published:** 2022-03-25

**Authors:** Delphine Bibbal, Philippe Ruiz, Panagiotis Sapountzis, Christine Mazuy-Cruchaudet, Estelle Loukiadis, Frédéric Auvray, Evelyne Forano, Hubert Brugère

**Affiliations:** ^1^IRSD, Université de Toulouse, INSERM, INRAE, ENVT, UPS, Toulouse, France; ^2^Université Clermont Auvergne, INRAE, UMR 454 MEDIS, Clermont-Ferrand, France; ^3^Université de Lyon, VetAgro Sup, National Reference Laboratory for E. coli (including VTEC), Marcy l'Etoile, France; ^4^Université de Lyon, Laboratoire d'Ecologie Microbienne de Lyon, CNRS, INRAE, Université de Lyon 1, VetAgro Sup, Microbial Ecology Laboratory, Research Group on Bacterial Opportunistic Pathogens and Environment, Villeurbanne, France

**Keywords:** *Escherichia coli*, EHEC, O157:H7, cattle, persistence, microbiota

## Abstract

Cattle are carriers, without clinical manifestations, of enterohemorrhagic *Escherichia coli* (EHEC) O157:H7 responsible for life-threatening infections in humans. A better identification of factors playing a role in maintaining persistence of such strains in cattle is required to develop more effective control measures. Hence, we conducted a study to identify farms with a persistent circulation of EHEC O157:H7. The EHEC O157:H7 herd status of 13 farms, which had previously provided bovine EHEC O157:H7 carriers at slaughter was investigated. Two farms were still housing positive young bulls, and this was true over a 1-year period. Only one fecal sample could be considered from a supershedder, and 60% of the carriers shed concentrations below 10 MPN/g. Moreover, EHEC O157:H7 represented minor subpopulations of *E. coli*. PFGE analysis of the EHEC O157:H7 strains showed that persistent circulation was due either to the persistence of a few predominant strains or to the repeated exposure of cattle to various strains. Finally, we compared fecal microbial communities of shedders (S) (*n* = 24) and non-shedders (NS) (*n* = 28), including 43 young bulls and nine cows, from one farm. Regarding alpha diversity, no significant difference between S *vs*. NS young bulls (*n* = 43) was observed. At the genus level, we identified 10 amplicon sequence variant (ASV) indicators of the S or NS groups. The bacterial indicators of S belonged to the family XIII UCG-001, *Slackia*, and *Campylobacter* genera, and *Ruminococcaceae* NK4A21A, *Lachnospiraceae*-UGC-010, and *Lachnospiraceae*-GCA-900066575 groups. The NS group indicator ASVs were affiliated to *Pirellulaceae*-1088-a5 gut group, *Anaerovibrio, Victivallis*, and *Sellimonas* genera. In conclusion, the characteristics enhancing the persistence of some predominant strains observed here should be explored further, and studies focused on mechanisms of competition among *E. coli* strains are also needed.

## Introduction

Enterohemorrhagic *Escherichia coli* (EHEC) cause life-threatening infections, which could lead to hemolytic uremic syndrome (HUS) in humans, particularly in children. The pathogenicity of EHEC is mainly due to the production of Shiga toxins (Stx) (1). Many EHEC serotypes have been associated with HUS cases, but EHEC O157:H7 is one of the main serotypes responsible for HUS cases in Europe and worldwide (2, 3). The main reservoir of EHEC is the digestive tract of ruminants. Various transmission routes of EHEC O157:H7 have been described, but the main one is the consumption of contaminated food and water (4). In particular, O157:H7 outbreaks were often associated with undercooked beef (5). The infectious dose of EHEC O157:H7 is very low as 10–100 colony-forming units (CFU) may induce symptoms (6). To date, no specific therapy exists to treat EHEC infections in humans. All these facts justify the implementation of preventive food safety interventions from primary production to consumption in order to provide food not contaminated by fecal EHEC O157:H7. In cattle production, control measures aim at reducing carriage and shedding of EHEC O157:H7 by cattle.

Preharvest food safety research is focused on the identification of intervention strategies aiming at reducing EHEC O157:H7 shedding, such as vaccines, probiotics, bacteriophages, sodium chlorate, and other feed additives. Certain intervention strategies showed some promise, but their impact remained limited to ensure the farm-to-table food safety continuum (7, 8). In addition, preharvest food safety research identified various factors driving shedding of EHEC O157:H7: seasonality, production system, diet, animal stress, and age (9). Investigations should, thus, be conducted to identify the mechanisms that result in persistent circulation of EHEC O157:H7 in cattle and their environment. Such studies could lead to the identification of a synergy of more effective control measures for the reduction of EHEC O157:H7 in cattle. Cattle that shed high levels of EHEC O157:H7 (i.e., above 10^4^ CFU/g of feces), called supershedder, appeared to have a substantial impact on the on-farm prevalence of this pathogen and transmission in the environment (10, 11). However, other factors might be considered to explain the persistence of EHEC O157:H7 involving strain, host, and environment-specific factors. Part of research exploring possible roles of these factors focused on a possible role of the digestive microbial communities. In particular, some differences in the fecal microbial communities were observed between O157:H7 supershedders and non-shedders, suggesting that there may be a relationship between the composition and diversity of the digestive microbiota and O157:H7 shedding (12, 13).

The objective of this study was to identify cattle farms with a persistent circulation of EHEC O157:H7 for a long period of time, in order to identify factors that might explain such a persistence. EHEC O157:H7 isolates from positive farms were characterized to evaluate if predominant strains play a significant role in this persistence. We also quantified the level of excretion of EHEC O157:H7 in order to evaluate whether supershedders contributed to this phenomenon. Finally, in one farm, we compared fecal microbial communities of EHEC O157:H7 shedders (S) and non-shedders (NS) in order to examine if the gastrointestinal ecosystem might affect EHEC persistence in a cattle farm.

## Materials and Methods

### Study Design

A previous prevalence study conducted in French slaughterhouses led to the identification of 17 farms that provided cattle that were positive for EHEC O157:H7 at slaughter (14). The objectives of the present study were to identify if EHEC O157:H7 shedding has persisted in these farms, and to explore factors that might play a role in the maintenance of O157:H7. Farmers were asked to participate on a voluntary basis and were assured of confidentiality. Two had ceased with cattle production and two did not want to participate. The study was, thus, conducted in 13 farms, named from A to M (Table 1). A first visit (spring/summer 2013) was conducted in order to identify the EHEC O157:H7 herd status. Twenty-one fecal samples were collected from young bulls and dairy cows in each farm (except for farms F and K, where only 20 and 14 samples could be collected, respectively). In the bovine species, young bulls and cows are, respectively, defined by the European legislation [Regulation (EU) No 1308/2013] as uncastrated males aged from 12 months to less to 24 months, and as females that have calved. One environmental sample per farm was also collected. Two farms were confirmed to be positive for EHEC O157:H7 and were consequently subjected to other herd visits. During 2013/2014, farms G and M were subjected to three and four supplementary herd visits, respectively (Table 1). Farms G and M housed about 440 and 160 animals (i.e., cows and young bulls), respectively.

**Table 1:** Number of fecal and environmental samples tested and number of positive samples per herd visit.

**Farm, ID herd visit (date of sampling)**	**No. of fecal samples**					**No. of environmental samples**	
	**Young bulls**			**Dairy cows**		
	**No. tested**	**No. of EHEC O157:H7-positive (%)**	**No. of EPEC O157:H7-positive (%)**	**No. tested**	**No. of EHEC O157:H7-positive**	**No. tested**	**No. of EHEC O157:H7-positive**
Farms A, B, C, D, E, F, H, I, J, K, L (spring/summer 2013)	60	0	0	163	0	11	0
Farm G							
1st (October 2013)	21	3 (14.3)	1 (4.8)	0	-	1	0
2nd (June 2014)	22	7 (31.8)	0 (0.0)	10	0	7	1
3rd (October 2014)	25	4 (16.0)	1 (4.0)	0	-	4	0
Farm M							
1st^t^ (July 2013)	21	7 (33.3)	0 (0.0)	0	-	1	0
2nd (October 2013)	21	3 (14.3)	3 (14.3)	0	-	1	0
3rd (April 2014)	24	13 (54.2)	0 (0.0)	10	0	7	1
4th (October 2014)	31	1 (3.2)	0 (0.0)	0	-	2	0

### Sample Collection

At each visit, fecal and environmental samples were collected. Feces were taken just after defecation. Environmental sampling was performed by attaching moistened gauze to the outside of disposable plastic boots (overshoe) and by walking 40 steps around the areas where the animals were kept, avoiding fecal pats. All samples were kept chilled and sent to the laboratory by overnight courier for analysis.

### Sample Enrichment and DNA Extraction for PCR-Based Screenings

Upon arrival, each fecal sample (10 g) was 10-fold diluted (w/v) in 90 ml of modified tryptone soya broth (Oxoid, Dardilly, France) supplemented with novobiocin (Oxoid, Dardilly, France) at 16 mg L^−1^ in Stomacher bags. Environmental samples (two pieces of gauze) were placed in Stomacher bags containing 225 ml of modified tryptone soya broth supplemented with novobiocin at 16 mg L^−1^. Stomacher bags were mixed and incubated overnight at 37°C. Bacterial DNA was extracted from 1 ml of each enriched broth using a lysis tube (Pall GeneDisc Technologies, Bruz, France), as already described (15).

### PCR-Based Screenings and Isolation Procedure

DNA extracts were subjected to a sequential real-time PCR-based approach for the detection of O157:H7 EHEC-associated genetic markers (*stx1, stx2, eae*-γ1, and *rfbE*_O157_) as previously described (14). Isolations were performed for samples that tested positive by PCR for the simultaneous presence of these markers. Isolation procedure consisted of immunomagnetic separation (IMS) assays using Dynabeads anti-*E.coli* O157 (Invitrogen, Cergy Pontoise, France), as recommended by the manufacturer. Ten microliters of immunoconcentrated bacteria were plated onto cefixime–tellurite–sorbitol–MacConkey (CT-SMAC) agar (Oxoid, Dardilly, France) and ChromoID O157:H7 agar (bioMérieux, Marcy l'Etoile, France). For each sample, five plates of each agar were plated, and all media were incubated overnight at 37°C. Up to 10 suspect colonies were tested by slide agglutination with serogroup-specific antisera (Statens Serum Institut, Copenhagen, Danemark). The serogroup O157 was confirmed by real-time PCR and the presence of *stx1, stx2*, and *eae*-γ1 was screened as described above. The presence of the *fliC*_H7_ alleles was also investigated as previously described (16). Isolates were also confirmed as *E. coli* using an API 20E test (bioMérieux, Marcy l'Etoile, France). Based on PCR results, *E. coli* isolates positive for *stx, eae*-γ1, *rfbE*_O157_, and *fliC*_H7_ genes were classified as Shiga toxin-producing EHEC O157:H7. *E. coli* isolates positive for *eae*-γ1, *rfbE*_O157_, and *fliC*_H7_, and negative for *stx* genes were classified as EPEC O157:H7.

### Enumeration of O157:H7 *Escherichia coli* and Generic *Escherichia coli*

Enumeration of EHEC O157:H7 was performed in a liquid medium by using a most probable number method. One gram of feces, stored at 4°C, was 10-fold serial diluted in tryptone salt broth (Oxoid, Dardilly, France). One milliliter of each dilution was added, in duplicate, to 9 ml of modified tryptone soya broth supplemented with novobiocin at 16 mg L^−1^. Broths were incubated overnight at 37°C. In order to isolate EHEC O157:H7 from positive (turbid) broths, IMS-based isolations were performed. Twenty-five microliters of immunoconcentrated bacteria were plated onto CT-SMAC agar and ChromoID O157:H7 agar. For each positive broth, five plates of each agar were plated, and all media were incubated overnight at 37°C. Suspect colonies were tested by PCR for the presence of *stx, eae*-γ1, and *rfbE*_O157_ genes. Enumeration was finally calculated from the number of EHEC O157:H7-positive duplicates or each dilution according to McCrady's tables, and expressed as the most probable number per gram of feces (MPN/g) (17). In order to perform generic *E. coli* counts, 10 g of feces was homogenized in 90 ml of saline solution, and 10-fold serial dilutions were prepared. Decimal dilutions were plated onto Petrifilm^TM^ Select *E. coli* (Grosseron, Saint Herblain, France). Enumerations were performed after incubation at 42°C during 24 h.

### Characterization of O157:H7 Enterohemorrhagic *Escherichia coli* and Enteropathogenic *Escherichia coli*

Subtyping of *stx*_1_ and *stx*_2_ genes was performed as previously described (18). The presence of additional EHEC-virulence markers (enterohemolysin *ehxA* gene and OI-122 associated genes, namely, *pagC, sen, efa1*, and *efa2* genes) was screened by PCR as previously described (19, 20). The presence of typical EPEC markers, i.e., *bfpA* and EPEC adherence factor (EAF) genes, was also tested by PCR (21, 22). *E. coli* isolates positive for the *eae* gene, and negative for the *bfpA* gene and EAF plasmid were classified as atypical enteropathogenic *E. coli* (aEPEC) (1). Finally, EHEC and EPEC strains were typed using the Standard PulseNet PFGE protocol for *E. coli* O157, as described previously (14).

### DNA Extraction, Amplification, and Sequencing for Fecal Bacteria Community Analysis

Samples were processed for DNA extraction and purification using the QIAamp DNA Stool Mini kit (Qiagen), following the manufacturer recommendations. The V4 region of the 16S rDNA gene was amplified and sequenced at the Génome Québec Innovation Center of McGill University (Montréal, Canada). The FastStart High Fidelity PCR System kit (Roche Diagnostics), together with primers 515F (5′-GTGCCAGCMGCCGCGGTAA-3′) and 806R (5′-GGACTACHVGGGTWTCTAAT-3′) were used to amplify the V4 region of the 16S rDNA gene (23). Sequencing was done using an Illumina MiSeq system (Illumina, USA). Resulting FASTQ files can be found in the NCBI Sequence Read Archive (SRA) under BioProject PRJNA599584.

### Sequencing Data Analysis

The 16S Illumina data have been analyzed by following the ≪Bioconductor Workflow for microbiome data analyses≫ (24) based on the dada2 algorithm developed to improve the detection of Amplicon Sequence Variants (ASVs) (25). The dada2 package has been used on R 3.5.1 (26) on a 32-core with 32-Gb RAM desktop computer for the pipeline's step of filtering, trimming, dereplication, to infer the sample composition, and to remove chimera. To improve the detection of rare variants from 16S amplicons and maintain a linear memory requirement, the dada2 procedure using the pseudo-pool algorithm of samples was used. The multiple-sequence alignment was performed using the DECIPHER R package (27). The Silva nr v.132 database (28) and a naïve Bayesian classifier (29) was used to assign taxonomy from kingdom to species.

### Statistical Analyses

The non-bacteria kingdom and singleton sequences (ASV, which only appears once) were removed before analyses. All the diversity analyses were performed with the Phyloseq R package (30). Raw ASV abundances were used for the alpha-diversity analyses. Beta-diversity analyses were performed using transformed abundance table with the DESeq2's variance stabilizing transformation (31, 32). Non-metric multidimensional scaling (NMDS) was used for the multivariate analysis of the community using the “vegan” package in R. Permutational multivariate analysis of variance (33) from the “vegan” R package were performed on ASV distance matrix to fit a regression model between the abundance distance matrix and variables. The microbiome differential abundance testing and log2foldChange estimate (34) were performed using the default multiple-inference correction of DESeq2 (Benjamini–Hochberg). Indicator species were identified using the “indicspecies” package in R (35, 36). We chose to use this method which focuses on identifying species that are both restricted to one group and with high fidelity (most samples in that group have the species), and thus, it offers a good complementary alternative method to the differential representation analysis using DESeq2. For this, a genus-level identity ASV table (similar to the one used for the DESeq2 analysis) was used as input. Each ASV ecological niche preference [shedders (S) or non-shedders (NS)]was identified using the Pearson's phi coefficient of association (corrected for unequal sample sizes) using the “indicspecies” package and 10,000 permutations. Only data from young bulls were used for all comparisons between S and NS to avoid confounding effects. All samples were considered as independent.

## Results

### Enterohemorrhagic *Escherichia coli* O157:H7 Herd Status

The presence of EHEC O157:H7 was screened in 13 farms (A to M), which provided cattle carrying EHEC O157:H7 at slaughterhouse 2 years before (14). The first visit allowed to identify two positive farms, namely, G and M, having three to seven young bulls shedding EHEC O157:H7 (Table 1). Two to three additional visits were performed in these two farms over a 1-year period, and each of them led to the detection of at least one shedder among young bulls. The percentage of young bulls shedding EHEC O157:H7 ranged from 14.3 to 31.8% and from 3.2 to 54.2% of the animals tested in farms G and M, respectively, depending on the time of visit. In each farm, an environmental sample led to the isolation of EHEC O157:H7 (during the second visit for farm G and the third visit for farm M). Finally, attempts to isolate EHEC O157:H7 also led to identification of young bulls harboring EPEC O157:H7 in farms G and M. None of the dairy cows tested from the farms visited in this study (including farms G and M) shed EHEC O157:H7 (Table 1).

### Virulence Profiles of Enterohemorrhagic *Escherichia coli* and Enteropathogenic *Escherichia coli* O157:H7

For farm G, the 11 EHEC O157:H7 strains isolated during the first and second visits carried the *stx*_1a_ subtype, whereas the four strains isolated during the third visit carried the *stx*_2c_ subtype (Table 2). For farm M, the 25 EHEC O157:H7 isolates possessed simultaneously the *stx*_1a_ and *stx*_2c_ subtypes (Table 3). All EHEC O157:H7 strains were positive for *eae*-γ1, enterohemolysin *ehx*A, and OI-122 associated genes (*pagC, sen, efa1*, and *efa2* genes). These genetic characteristics were shared by EPEC O157:H7 isolates (two from farm G and three from farm M), except that these EPEC lacked the *stx* genes (Tables 2, 3). All EPEC were negative for *bfp* and EPEC adherence factor (EAF) plasmid, thereby justifying their classification as atypical EPEC (aEPEC).

**Table 2:** Origin, virulence profiles, counts, and PFGE types (PT) of EHEC and EPEC O157:H7 isolated from fecal or environmental samples and counts of generic *Escherichia coli* in farm G.

**Herd visit**	**Bovine ID**	**Category^a^**	**Strain ID**	**Presence of genes** ^b^ **:**			**EHEC O157:H7 counts^c^ (MPN/g)**	**Generic *E. coli* counts^**c**^ (CFU/g)**	**PT**
				***stx1* (subtype)**	***stx2* (subtype)**	***eae* (subtype)**			
1	1-b1	YDB	1biss1-1	+ (*stx*_1a_)	-	+ (γ1)	<6.0E + 00	n.d.	23
1	1-b18	YDB	1biss18-1	+ (*stx*_1a_)	-	+ (γ1)	<6.0E + 00	n.d.	30
1	1-b20	YDB	1biss20-1	+ (*stx*_1a_)	-	+ (γ1)	<6.0E + 00	n.d.	22
1	1-b20	YDB	1biss20-6	-	-	+ (γ1)	n.a.	n.d.	16
2	3-G2	YDB	3G2-1	+ (*stx*_1a_)	-	+ (γ1)	6.0E + 01	4.5E + 06	19
2	3-G5	YDB	3G5-1	+ (*stx*_1a_)	-	+ (γ1)	6.0E + 00	7.0E + 05	17
2	3-G6	YDB	3G6-1	+ (*stx*_1a_)	-	+ (γ1)	1.3E + 01	2.3E + 06	21
2	3-G7	YDB	3G7-1	+ (*stx*_1a_)	-	+ (γ1)	<6.0E + 00	2.2E + 06	29
2	3-G8	YDB	3G8-1	+ (*stx*_1a_)	-	+ (γ1)	<6.0E + 00	4.0E + 06	24
2	3-G13	YDB	3G13-1	+ (*stx*_1a_)	-	+ (γ1)	<6.0E + 00	1.4E + 06	26
2	3-G14	YDB	3G14-1	+ (*stx*_1a_)	-	+ (γ1)	2.5E + 02	2.4E + 06	18
2	-	E	3GP5-1	+ (*stx*_1a_)	-	+ (γ1)	n.a.	n.d.	20
3	4-G2	YDB	4G2-1	-	+ (*stx*_2c_)	+ (γ1)	2.5E + 02	5.9E + 05	28
3	4-G11	YDB	4G11-1	-	+ (*stx*_2c_)	+ (γ1)	6.0E + 00	2.4E + 05	15
3	4-G12	YDB	4G12-1	-	-	+ (γ1)	n.a.	n.d.	14
3	4-G19	YDB	4G19-1	-	+ (*stx*_2c_)	+ (γ1)	n.d.	n.d.	13
3	4-G23	YDB	4G23-1	-	+ (*stx*_2c_)	+ (γ1)	n.d.	n.d.	13

a *YBB, young beef bull; YDB, young dairy bull; E, environmental sample*.

b *+, detected by PCR; -, not detected by PCR*.

c *n.a., not applicable; n.d., not determined*.

**Table 3:** Origin, virulence profiles, counts, and PFGE types (PT) of enterohemorrhagic *Escherichia coli* (EHEC) and enteropathogenic *Escherichia coli* (EPEC) O157:H7 isolated from fecal or environmental samples and counts of generic *Escherichia coli* in farm M.

**Herd visit**	**Bovine ID**	**Category^a^**	**Strain ID**	**Presence of genes** ^b^ **:**			**EHEC O15.7:H7 counts^c^ (MPN/g)**	**Generic *E. coli* counts^c^ (CFU/g)**	**PT**
				***stx1* (subtype)**	***stx2* (subtype)**	***eae* (subtype)**			
1	1-M3	YBB	1M3-1	+ (*stx*_1a_)	+ (*stx*_2c_)	+ (γ1)	1.3E + 03	n.d.	5
1	1-M5	YBB	1M5-1	+ (*stx*_1a_)	+ (*stx*_2c_)	+ (γ1)	1.3E + 01	n.d.	11
1	1-M6	YDB	1M6-1	+ (*stx*_1a_)	+ (*stx*_2c_)	+ (γ1)	6.0E + 01	n.d.	5
1	1-M7	YDB	1M7-1	+ (*stx*_1a_)	+ (*stx*_2c_)	+ (γ1)	2.5E + 02	n.d.	7
1	1-M9	YBB	1M9-1	+ (*stx*_1a_)	+ (*stx*_2c_)	+ (γ1)	6.0E + 01	n.d.	8
1	1-M17	YDB	1M17-1	+ (*stx*_1a_)	+ (*stx*_2c_)	+ (γ1)	2.5E + 02	n.d.	6
1	1-M19	YDB	1M19-1	+ (*stx*_1a_)	+ (*stx*_2c_)	+ (γ1)	<6.0E + 00	n.d.	5
2	1b-P1	YBB	1bisP1-1	+ (*stx*_1a_)	+ (*stx*_2c_)	+ (γ1)	<6.0E + 00	n.d.	31
2	1b-P2	YDB	1bisP2-1	-	-	+ (γ1)	n.a.	n.d.	3
2	1b-P4	YBB	1bisP4-1	-	-	+ (γ1)	n.a.	n.d.	27
2	1b-P5	YBB	1bisP5-1	+ (*stx*_1a_)	+ (*stx*_2c_)	+ (γ1)	1.3E + 04	n.d.	8
2	1b-P16	YBB	1bisP16-1	-	-	+ (γ1)	n.a.	n.d.	4
2	1b-P18	YDB	1bisP18-1	+ (*stx*_1a_)	+ (*stx*_2c_)	+ (γ1)	2.5E + 02	n.d.	12
3	2-M9	YBB	2M9-1	+ (*stx*_1a_)	+ (*stx*_2c_)	+ (γ1)	<6.0E + 00	8.00E + 06	12
3	2-M11	YBB	2M11-1	+ (*stx*_1a_)	+ (*stx*_2c_)	+ (γ1)	n.d.	n.d.	1
3	2-M12	YBB	2M12-1	+ (*stx*_1a_)	+ (*stx*_2c_)	+ (γ1)	6.0E + 00	4.90E + 07	2
3	2-M13	YBB	2M13-1	+ (*stx*_1a_)	+ (*stx*_2c_)	+ (γ1)	2.5E + 02	1.70E + 07	9
3	2-M14	YBB	2M14-1	+ (*stx*_1a_)	+ (*stx*_2c_)	+ (γ1)	<6.0E + 00	2.10E + 07	1
3	2-M15	YBB	2M15-1	+ (*stx*_1a_)	+ (*stx*_2c_)	+ (γ1)	<6.0E + 00	4.50E + 06	7
3	2-M16	YBB	2M16-1	+ (*stx*_1a_)	+ (*stx*_2c_)	+ (γ1)	<6.0E + 00	1.30E + 06	1
3	2-M17	YBB	2M17-1	+ (*stx*_1a_)	+ (*stx*_2c_)	+ (γ1)	2.5E + 01	2.10E + 06	1
3	2-M18	YBB	2M18-1	+ (*stx*_1a_)	+ (*stx*_2c_)	+ (γ1)	<6.0E + 00	4.40E + 06	10
3	2-M19	YBB	2M19-1	+ (*stx*_1a_)	+ (*stx*_2c_)	+ (γ1)	6.0E + 00	6.00E + 05	1
3	2-M21	YBB	2M21-1	+ (*stx*_1a_)	+ (*stx*_2c_)	+ (γ1)	<6.0E + 00	1.00E + 05	5
3	2-M22	YBB	2M22-1	+ (*stx*_1a_)	+ (*stx*_2c_)	+ (γ1)	<6.0E + 00	2.90E + 06	1
3	2-M24	YBB	2M24-1	+ (*stx*_1a_)	+ (*stx*_2c_)	+ (γ1)	<6.0E + 00	1.20E + 06	1
3	-	E	2MP4-1	+ (*stx*_1a_)	+ (*stx*_2c_)	+ (γ1)	n.a.	nd	1
4	4-M30	YBB	4m30-1	+ (*stx*_1a_)	+ (*stx*_2c_)	+ (γ1)	<6.0E + 00	2.80E + 05	25

a *YBB, young beef null, YDB; young dairy bull; E, environmental sample*.

b *+, detected by PCR; -, not detected by PCR*.

c *n.a., not applicable; n.d., not determined*.

### Counts of Enterohemorrhagic *Escherichia coli* O157:H7 and Generic *Escherichia coli*

EHEC O157:H7 counts were low (Tables 2, 3). These counts were below 10 MPN/g of feces in 60% of the young bulls. Only one young bull from farm M (second visit) showed an EHEC count above 10^4^ MPN/g of feces and could be considered as a supershedder (Table 3). By contrast, generic *E. coli* counts ranged from to 1 × 10^5^ to 4.9 × 10^7^ CFU/g of feces indicating that EHEC O157:H7 represented minor subpopulations of *E. coli*.

### Diversity of Enterohemorrhagic *Escherichia coli* O157:H7 Isolates

The 40 EHEC and 5 aEPEC O157:H7 isolated from farms G and M were subtyped by pulse-field gel electrophoresis (PFGE) (Tables 2, 3 and Supplementary Figure 1). Two additional EHEC O157:H7 strains isolated 2 years before in the same two farms were added to the dendrogram (see Supplementary Figure 1). With regard to farm M, 23 EHEC O157:H7 and 2 EPEC O157:H7 showed high genetic relatedness as most of them clustered together and displayed PFGE types (PT) (referred to as PT 1 to PT 12) with more than 82% similarity (Supplementary Figure 1). Four other strains, including the EHEC O157:H7 strain isolated in 2010, did not belong to this cluster. Among the cluster, several strains shared the same PT, including strains isolated from different animals indicating transmission between these animals or exposure to a common source. Some strains with an identical PT were also isolated from different herd visits illustrating their persistence over time in farm M (Table 3). Strain 1bisP5-1 isolated during the second visit from a supershedder young bull (i.e., 1.3 × 10^4^ MPN/g) displayed the same PT (PT 8) as strain 1M9-1 shed during the first visit at a lower level (i.e., 60 MPN/g). Strain 2MP4-1 isolated from an environmental sample displayed the same PT (PT 1) as strains isolated from seven young bulls. With regard to farm G, a higher diversity of PFGE profiles was observed (Supplementary Figure 1). Of the 18 O157:H7 strains isolated from this farm, only two strains shared the same PT (PT 13), and these were isolated during the same visit (Table 2).

### Diversity and Taxonomic Analysis of the Fecal Microbiota of Animals From Farm M

Fecal samples from 52 animals corresponding to 9 dairy cows (C) and 43 young bulls (YB), including 33 beef bulls and 10 dairy bulls, from farm M were analyzed by 16S rDNA amplicon MiSeq sequencing. These included the samples of 24 young bulls shedding EHEC O157:H7 (S) (Table 3) and the samples of 28 non-shedders (NS) consisting of 16 young beef bulls, 9 dairy cows, and 3 young bulls shedding EPEC O157:H7. The 16S rDNA amplicon analysis of the fecal samples generated 4,757,143 high-quality sequences, with an average of 179,514 sequences per sample after quality filtering. The overall number of ASVs detected by analysis was 7,264, based on a 97% nucleotide sequence identity between reads and after removing *Archaea* (37) and non-attributed (5) sequences. Rarefaction curves showed that the sampling was sufficient for all the 52 samples (not shown).

**Figure 1:**
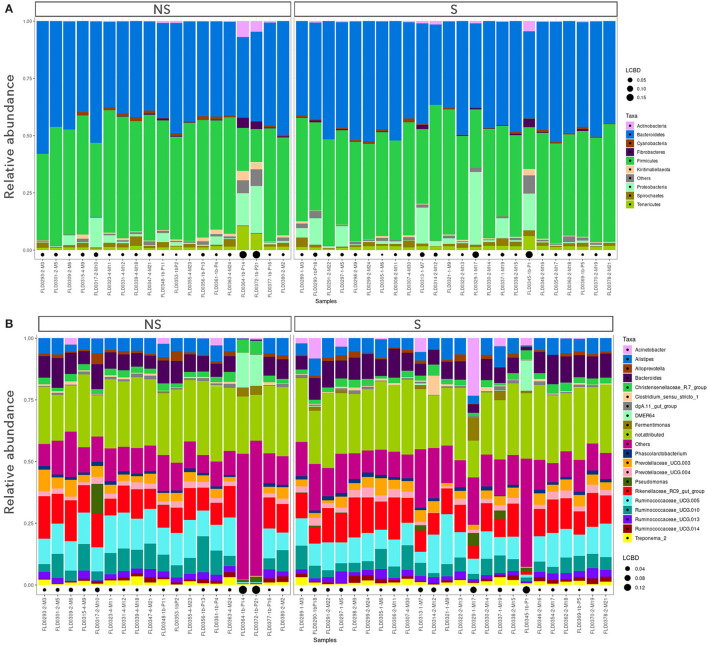
Taxonomic profiles of the fecal bacteria from enterohemorrhagic *Escherichia coli* (EHEC) O157:H7 shedders (S) and non-shedders (NS). **(A)** Bar plot representation of relative proportion of the nine major phyla in the fecal samples. **(B)** Bar plot representation of relative proportion of the 20 major genera in the fecal samples. Only data from young bulls were used for this analysis. Black points size is proportional to the local contribution to beta diversity for each sample.

**Figure 2:**
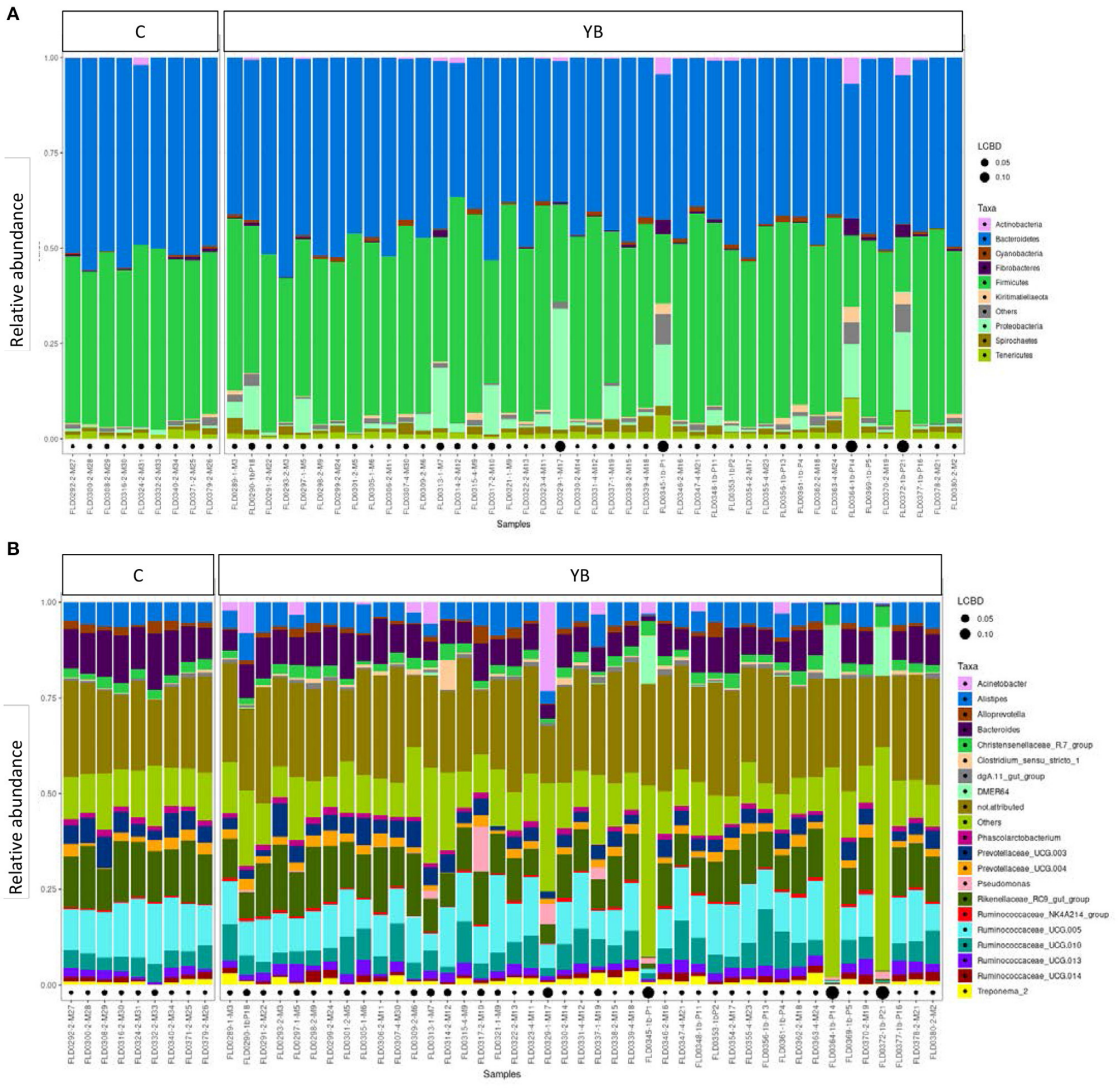
Taxonomic profiles of the fecal bacteria from dairy cows (C) and young bulls (YB). **(A)** Bar plot representation of relative proportion of the nine major phyla in the fecal samples. **(B)** Bar plot representation of relative proportion of the 20 major genera in the fecal samples. Black points size is proportional to the local contribution to beta diversity for each sample.

To compare the microbiota of S *vs*. NS, only the data from young bulls (43 samples) were used, in order to avoid confounding effects. Alpha diversity indices are shown in Supplementary Figure 2. By repeated measure of analysis of variance (ANOVA), it was apparent that there were no significant differences in alpha diversity between S and NS (43 samples) or between YB and C (52 samples) (*p* > 0.1). We analyzed the relative abundance of bacteria present in the feces of the animals. Animals were grouped by S *vs*. NS (Figure 1), and by YB *vs*. C (Figure 2). At the phylum, family, and genus level, there were no obvious differences between groups. The major phyla found in all groups were *Bacteroidetes, Firmicutes*, and *Proteobacteria* (Figures 1, 2). While the relative abundance of *Bacteroidetes* varied from 35 to 57% depending on the animals, with a mean at 45.9%, that of *Firmicutes* was more variable, from 14.5 to 59.2%. *Proteobacteria* relative abundance was also highly variable, from 0.1 to 31.8% depending on the animals. Five animals with a very high proportion of *Proteobacteria* (1-M17, 1b-P21, 1b-P1, 1-M7, and 1b-P14) were also those with the lowest proportion of *Firmicutes* and were all young bulls (Figures 1A, 2A). Three of these animals were EHEC O157:H7 shedders (1-M17, 1b-P1, and 1-M7). In samples from animals 1b-P1, 1b-P14, and 1b-P21, the relative abundance of *Tenericutes* was also higher than for the other animals (from 6 to 10%). Analysis at the genus level (Figures 1B, 2B) showed that in animals with a high proportion of *Proteobacteria*, this phylum was mostly composed of *Pseudomonas, Acinetobacter, Thauera*, or non-attributed genera. In these samples, the *Enterobacteriaceae* family was not represented (not shown).

### Overall Differences in Community Structure and Differentially Represented Amplicon Sequence Variants

Overall differences in community structure were assessed using NMDS (Supplementary Figure 3). No clear separation of samples was observed for bacteria associated with S and NS or C and YB. We further examined the differences in bacterial community structure between S and NS samples (43 YB samples) using Permanova (on Bray Curtis distance), which showed that samples did not cluster by shedding status (*p* > 0.05). However, when adding the sampling time (farm visit) as variable, the community structure appeared different between S and NS feces (*p* = 0.0496). In addition, the sampling time had clearly a significant effect on the microbiota beta diversity (*p* = 0.0001) but not on alpha diversity (not shown).

The analysis of ASVs differential abundance between YB and C fecal samples (using DESeq2) identified 24 significantly differential ASVs (Supplementary Figure 4), but the DESeq2 analysis did not find differentially abundant ASVs between S and NS. However, using the indicator analysis “indicspecies” package, we identified six ASVs as significant indicators of the S group, and four ASVs as indicators of the NS group (Table 4). Six of these indicator species belong to the *Firmicutes* phylum. The bacterial indicators of S belong to family XIII UCG-001, *Slackia*, and *Campylobacter* genera, and *Ruminococcaceae* NK4A21A, *Lachnospiraceae*-UGC-010, and *Lachnospiraceae*-GCA-900066575 groups. The NS group indicator ASVs were affiliated to *Pirellulaceae*-1088-a5 gut group, *Anaerovibrio, Victivallis*, and *Sellimonas* genera.

**Table 4:** Indicator species analysis comparing EHEC O157:H7 shedder (S) and non-shedder (NS) groups.

**EHEC O157:H7**	**Phylum**	**Family**	**Genus**	**Corr**.	***P-*value**
**status^a^**				**index**	
S	*Firmicutes*	Family_XIII	Family_XIII_UCG-001	0.488	0.001
S	*Actinobacteria*	*Eggerthellaceae*	*Slackia*	0.318	0.024
S	*Epsilonproteobacteria*	*Campylobacteraceae*	*Campylobacter*	0.327	0.024
S	*Firmicutes*	*Lachnospiraceae*	*Lachnospiraceae*_UCG-010	0.301	0.049
S	*Firmicutes*	*Ruminococcaceae*	*Ruminococcaceae*_NK4A214_group	0.324	0.035
S	*Firmicutes*	*Lachnospiraceae*	*Lachnospiraceae*_GCA-900066575	0.301	0.024
NS	*Planctomycetes*	*Pirellulaceae*	*Pirellulaceae*-1088-a5_gut_group	0.326	0.034
NS	*Firmicutes*	*Veillonellaceae*	*Anaerovibrio*	0.323	0.034
NS	*Lentisphaerae*	*Victivallaceae*	*Victivallis*	0.259	0.012
NS	*Firmicutes*	*Lachnospiraceae*	*Sellimonas*	0.296	0.036

a *S, EHEC O157:H7 shedder; NS, EHEC O157:H7 non-shedder*.

## Discussion

The objective of this study was to explore factors involved in the persistence of EHEC O157:H7 in cattle farms. To this aim, two farms (G and M) showing the persistent presence of EHEC O157:H7 were identified among 13 farms that provided cattle carrying EHEC O157:H7 at slaughterhouse 2 years before (14).

### Characteristics of Enterohemorrhagic *Escherichia coli* O157:H7 Isolated Strains

EHEC O157:H7 were isolated from young bulls, but never from cows. This is in agreement with other studies showing that younger animals are associated with an increased risk of shedding EHEC O157:H7 (38, 39). Moreover, the fact that EHEC O157:H7 strains were isolated from only a fraction of young bulls tested at each visit suggests that some positive animals might have been missed due to certain limitations of our studies related to the sporadic pattern and short duration of EHEC O157:H7 fecal shedding by animals. Indeed, a growing number of longitudinal studies have led to the hypothesis that *E. coli* O157 prevalence may be heterogeneous (40–42). Finally, two EHEC O157:H7 strains were isolated from the farm environment, including one strain in farm M that displayed the same PFGE type as several isolates retrieved from fecal samples. As already proposed by others, our results suggest that the analysis of environmental samples might be useful to determine the herd status with respect to EHEC shedding by animals (43). All EHEC O157:H7 strains isolated were positive for *stx*_1a_ and/or *stx*_2c_. Additionally, they were all positive for the *eae*-γ1 gene, the enterohemolysin *ehxA* gene, and OI-122-associated genes. These EHEC virulence markers have already been shown to be associated with O157:H7 strains that cause severe outbreaks (19, 44).

### Enterohemorrhagic *Escherichia coli* O157:H7 Fecal Concentrations Were Low

As EHEC O157:H7 supershedders have been identified to play a key role in the transmission of EHEC O157:H7 at the farm level (10), we performed counts of EHEC O157:H7 to identify such animals. For 60% of the shedders, these counts were very low (≤10 MPN/g of feces), and only one animal was a supershedder. This is in agreement with longitudinal studies that identified low numbers of supershedders in the herds examined (11, 45, 46). Interestingly, the strain from the supershedder shared the same PT as one strain shed in an earlier visit at a low concentration, suggesting that strain-specific characteristics are not sufficient for causing supershedding by cattle, as observed previously (47). Finally, the comparison of the EHEC O157:H7 counts to the generic *E. coli* counts from the same animals showed that EHEC O157:H7 represented a minority of the *E. coli* population. This observation confirmed the interest of researches focused on mechanisms of competition among *E. coli* strains in order to identify factors controlling the proliferation of EHEC in the digestive tract, such as colicins, for example (48).

### Persistence of Enterohemorrhagic *Escherichia coli* O157:H7 in Farms Was Due Either to the Circulation of Some Predominant Strains or to the Exposure of Cattle to Various Strains

Further characterization of EHEC O157:H7 strains revealed different patterns of shedding dynamics in the two farms investigated. In farm M, the 25 EHEC O157:H7 strains isolated during four herd visits showed identical virulence profiles. Notably, they were all positive for the *stx*_1a_ and *stx*_2c_ subtypes. PFGE analysis revealed that the majority of these strains were highly related and clustered together, including strains with either identical PT or showing 82% of similarity. Although up to five band losses have been reported following subculturing in the laboratory (49), only a few studies have considered the stability of PFGE profiles following host passage or exposure to environmental stimuli. Yoshii N. et al. reported a one to six band loss following PFGE analysis of *E. coli* O157:H7 after calf inoculation (50). In the light of these elements, our results showed that the *E. coli* O157:H7 isolates were highly related, suggesting they might originate from the same strain. Nevertheless, two EHEC O157:H7 strains from farm M belonged to PT quite different from that of the clustered isolates suggesting the circulation, although at a lower extent, of additional EHEC O157:H7 strains within this farm. The situation in farm G differed from that in farm M on several aspects. First, strains carrying different *stx* subtypes were isolated, including *stx*_1a_-positive strains during the two first visits followed by *stx*_2c_-positive strains during the last visit. Second, these strains showed a high diversity by PFGE analysis. Altogether, these results indicated that a higher number of EHEC O157:H7 strains that are unrelated circulated within this farm. The persistence of an O157:H7-positive herd status was, therefore, related to distinct events as observed by others (40, 42, 51, 52). On the one hand, it may result from continuous exposure of cattle to various strains as observed in farm G and, to a lower extent, in farm M. This was also the case for both farms G and M when sampled 2 years apart as the two strains isolated previously at slaughter were not the same as those isolated here. On the other hand, persistence of a positive herd status may be caused by the circulation of a predominant strain (and its derivatives resulting from genetic variations associated with minor PFGE variations) as observed in farm M. One strain (2M9-1) belonging to the cluster of related strains from farm M, was subjected to further studies in order to explore factors involved in the persistence (53). Our results showed that this strain (renamed MC2) had a large number of colonization factors known to bind to intestinal epithelium and to biotic or abiotic surfaces. We also showed that MC2 had the capacity to produce biofilms and to activate stress fitness genes in bovine feces. Taken together, these results could explain the persistence of this strain and its derivatives in the farm environment, acting as a source of contamination for cattle.

### Differences in the Fecal Microbial Communities of Enterohemorrhagic *Escherichia coli* O157:H7 Shedders and Non-shedders

Although different factors have been associated with EHEC shedding (37), several studies have suggested that microbiota composition plays a critical role in the establishment and/or ecology of *E*. *coli* O157:H7 within the intestinal tract of cattle. Some recent results supported this hypothesis (12, 13, 54–57), but the results differ between studies and the genera associated with the fecal microbiota of EHEC shedders and non-shedders varied from one study to another (58). In the present study, when analyzing the young bulls' fecal microbiota, we did not find any significant difference in alpha diversity between EHEC O157:H7 shedders *vs*. non-shedders. Furthermore, there was no significant difference in the overall community structure of these two groups, but the sampling time had a clear effect on the community structure. The indicator species analysis identified 10 indicators of the S or NS groups. Six ASVs were indicators of the S group, including four sequences affiliated to the *Firmicutes* phylum (two *Lachnospiracea*, one *Ruminococcacea*, and one Family XIII). Four ASVs were indicators of the NS group, including two *Firmicutes* sequences (*Lachnospiracea* and *Veillonellacea*), one *Planctomycetes*, and one *Lentisphaerae* ASVs. Identifying genera associated with non-shedding status could help in the design of potential new direct-fed microbial candidates to limit cattle STEC shedding (58).

In a previous study on 240 calves, the Shannon diversity index was lower in fecal samples from animals colonized by EHEC (54). In another study on 40 dairy cattle, the average microbiota richness of cows shedding EHEC was also lower than that of non-shedders, but statistical analysis did not find differentially abundant taxa between them (55). In a similar study on feedlot steers, Xu and colleagues found that supershedders exhibited higher bacterial richness and diversity than non-shedders, and 72 OTUs were found as differentially abundant between the two groups (13). In another work on feedlot steers, the overall bacterial community structure did not differ by *E*. *coli* O157:H7 shedding status, but 4 OTUs (*Ruminococcus, Selenomonas, Campylobacter*, and *Streptococcus* genera) were more abundant in supershedders (57). *Campylobacter* was also found to have a stronger preference for the gastrointestinal tract (GIT) of EHEC shedders in our work. A recent study on several farms in the USA showed that the fecal microbiota of farms with a high-STEC prevalence had greater richness compared with those of farms with a low-STEC prevalence, but no alpha diversity difference was observed when comparing EHEC shedders (*stx* and *eae* positive) and controls (59). These authors found 30 genera to be differentially abundant between STEC carriers and non-carriers, which were different from the ones identified in the present work. Wang and colleagues compared the mucosal microbiota of the rectoanal junction of supershedders and non-shedders, and they identified OTUs unique to SS or NS (56), but none of them was similar to the ones identified in the present study. As illustrated by these examples, correlating EHEC-shedding status with fecal or intestinal microbiota composition, richness, or specific taxa abundance is not straightforward. Indeed, the inconsistency in the results may reflect the variability in the inter-animal microbiota composition, which may be due to several factors including the animal age (54, 60) or diet (12, 59), but also to the farm environment or the methodology used in the different analyses. It is also known that the season has a strong impact on the shedding status of cattle, and a seasonal effect has also been shown on the microbiota composition of the GIT (feces) (9, 61, 62). Further studies should therefore compare the microbiota of a high number of animals of the same age and diet, and similar rearing mode. Finally, our study also compared the fecal microbiota of young bulls *vs*. lactating cows, and found 24 genera differentially abundant between these two groups. Although several studies monitored the evolution of the fecal microbiota of calves from birth to weaning (60, 63–65), only a few of them compared young bulls and lactating cows. Dill-Mcfarland et al. showed that the bacterial fecal community of 1- and 2-year-old animals, clustered clearly differently from that of younger ones (2–8 months) (60). As in our study, the genus *Faecalibacterium* was found much more abundant in feces of young animals.

## Conclusion

In conclusion, our study allowed to identify two farms with persistent EHEC O157:H7-positive status. The concentrations of such strains were low in fecal samples from shedders, suggesting that supershedding did not play a major role in the persistence of EHEC O157:H7 in these two farms. PFGE analysis revealed that the persistence of EHEC O157:H7 in cattle farms was either due to the persistent circulation of a limited set of well-established predominant strains or to the exposure of cattle to various genetically unrelated strains. Diversity and taxonomic analysis of the fecal microbiota did not reveal major differences between shedders and non-shedders, but identified 10 ASV indicators of the shedding status. Other work from our group focused on the factors involved in the persistence of the EHEC O157:H7 strain MC2, belonging to a cluster of related persistent strains, in bovine feces and gastrointestinal contents (53). Further studies performed in cattle experimentally challenged with such strains are now required. By minimizing the variabilities of a field study, these studies might allow the investigation not only of the dynamics of ecology of such strains along the digestive tract of cattle but also the investigation of animal factors playing a role in persistence. Finally, our study confirmed that the mechanisms of competitive inhibition among *E. coli* populations should be investigated in order to identify mechanisms resulting in differential proliferation of EHEC O157:H7.

## Data Availability Statement

The datasets presented in this study can be found in online repositories. The names of the repository/repositories and accession number(s) can be found at: https://www.ncbi.nlm.nih.gov/, bioproject/PRJNA599584.

## Author Contributions

DB, EL, FA, and HB conceived and designed the project. DB and EF wrote the manuscript. EL and FA reviewed the manuscript. CM-C and EL performed the PFGE experiment. PR, PS, and EF performed the microbiota analysis. All authors have read and agreed to the published version of the manuscript.

## Funding

This work was supported by funds from the French Cattle and Meat Association (Interbev), the French National Authority for Agriculture and Sea Products (FranceAgriMer) (n SECU-11-005), the French Ministry of Agriculture and the Agence Nationale de la Recherche (research grant ANR-21-CE21-0006-01).

## Conflict of Interest

The authors declare that the research was conducted in the absence of any commercial or financial relationships that could be construed as a potential conflict of interest.

## Publisher's Note

All claims expressed in this article are solely those of the authors and do not necessarily represent those of their affiliated organizations, or those of the publisher, the editors and the reviewers. Any product that may be evaluated in this article, or claim that may be made by its manufacturer, is not guaranteed or endorsed by the publisher.
