# Supplementary material for: Persistent Circulation of Enterohemorrhagic Escherichia coli (EHEC) O157:H7 in Cattle Farms: Characterization of Enterohemorrhagic Escherichia coli O157:H7 Strains and Fecal Microbial Communities of Bovine Shedders and Non-shedders
Source: Front Vet Sci. 2022 Mar 25;9:852475. doi: 10.3389/fvets.2022.852475 (PMC8994043; doi:10.3389/fvets.2022.852475)
Supplement: Supplementary file 1 [file Data_Sheet_1.docx]

**SUPPLEMENTARY FIGURES**


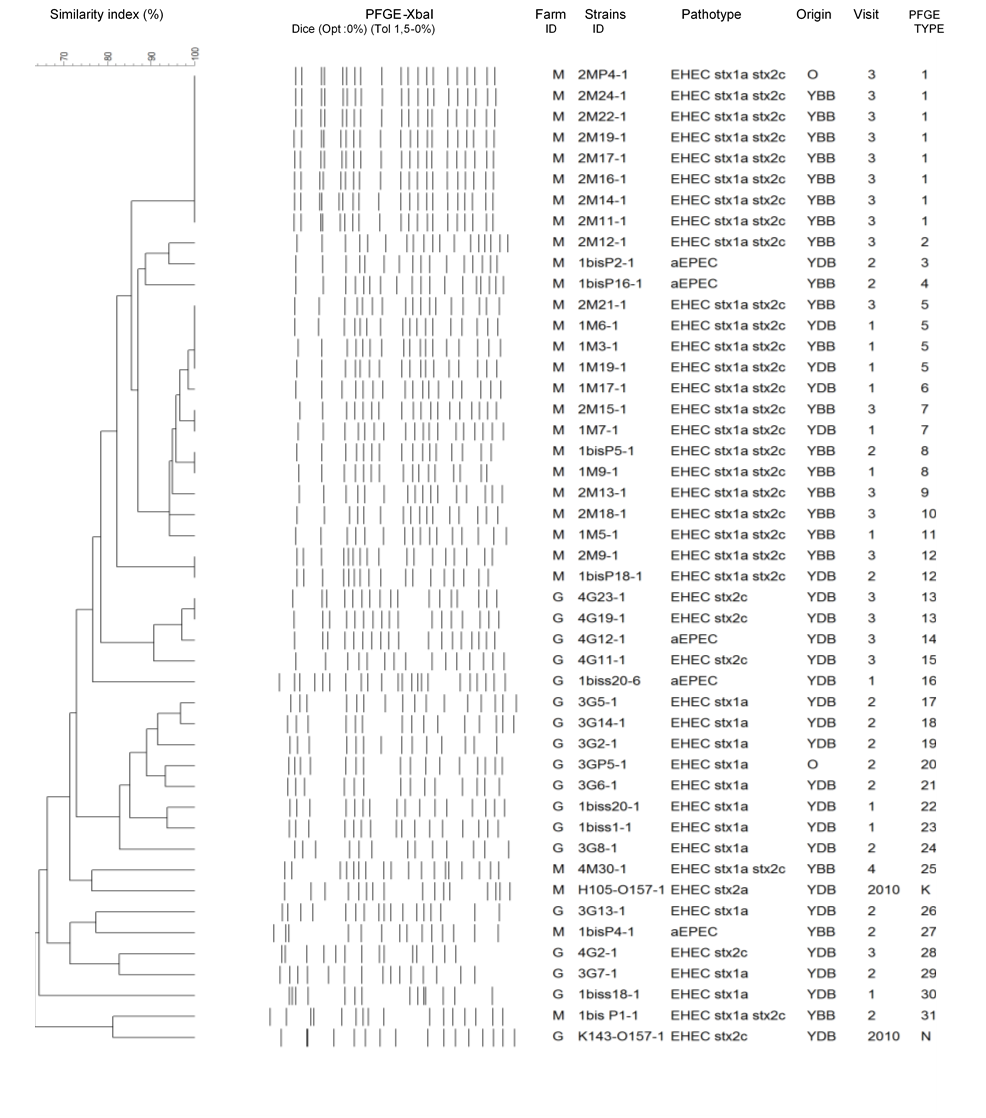
**Supplementary Figure 1.** XbaI PFGE patterns and origins of 45 EHEC and EPEC O157:H7 strains isolated in farm M and G in France and 2 EHEC O157:H7 strains (H105-O157-1 and K143-O157-1) isolated from the same farms two years earlier. The dendrogram was generated using the band-based Dice similarity coefﬁcient with a 1.5% band position tolerance and the unweighted pair group method with arithmetic mean clustering. YBB: Young Beef Bull, YDB: Young Dairy Bull; O: Overshoe.


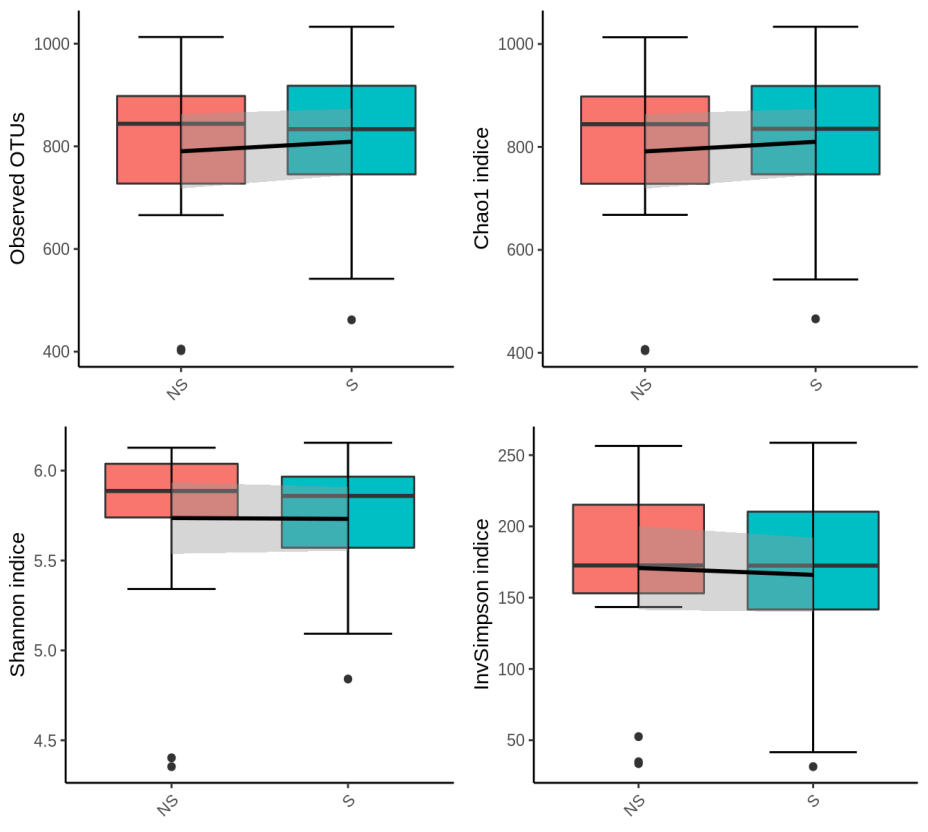


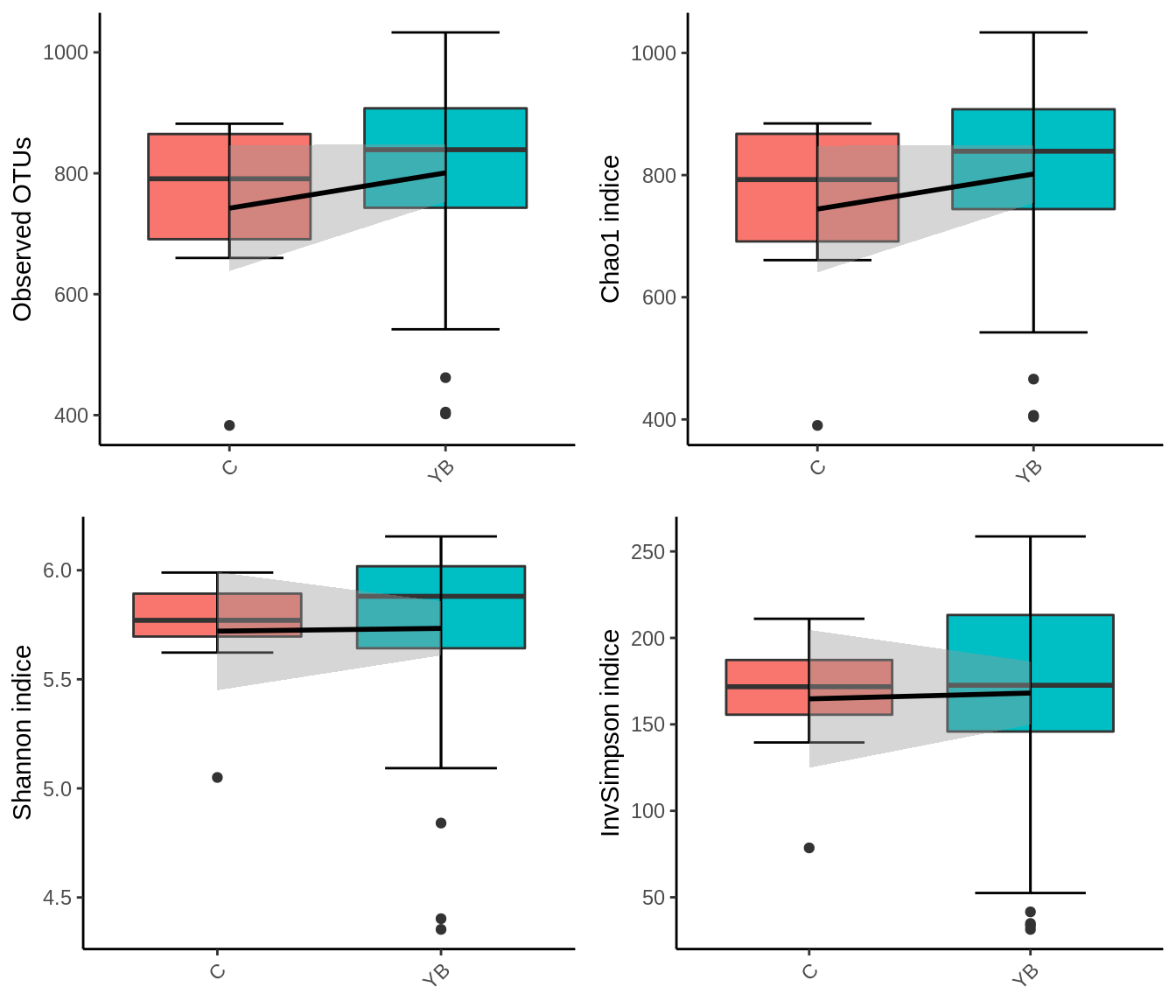


**A**

**B**

**Supplementary Figure 2.** Diversity indices of microbiota in fecal samples from EHEC O157:H7 non-shedders (NS) and shedders (S), and cows (C) and young bulls (YB). Only data from young bulls (43 samples) were used for comparing S and NS.


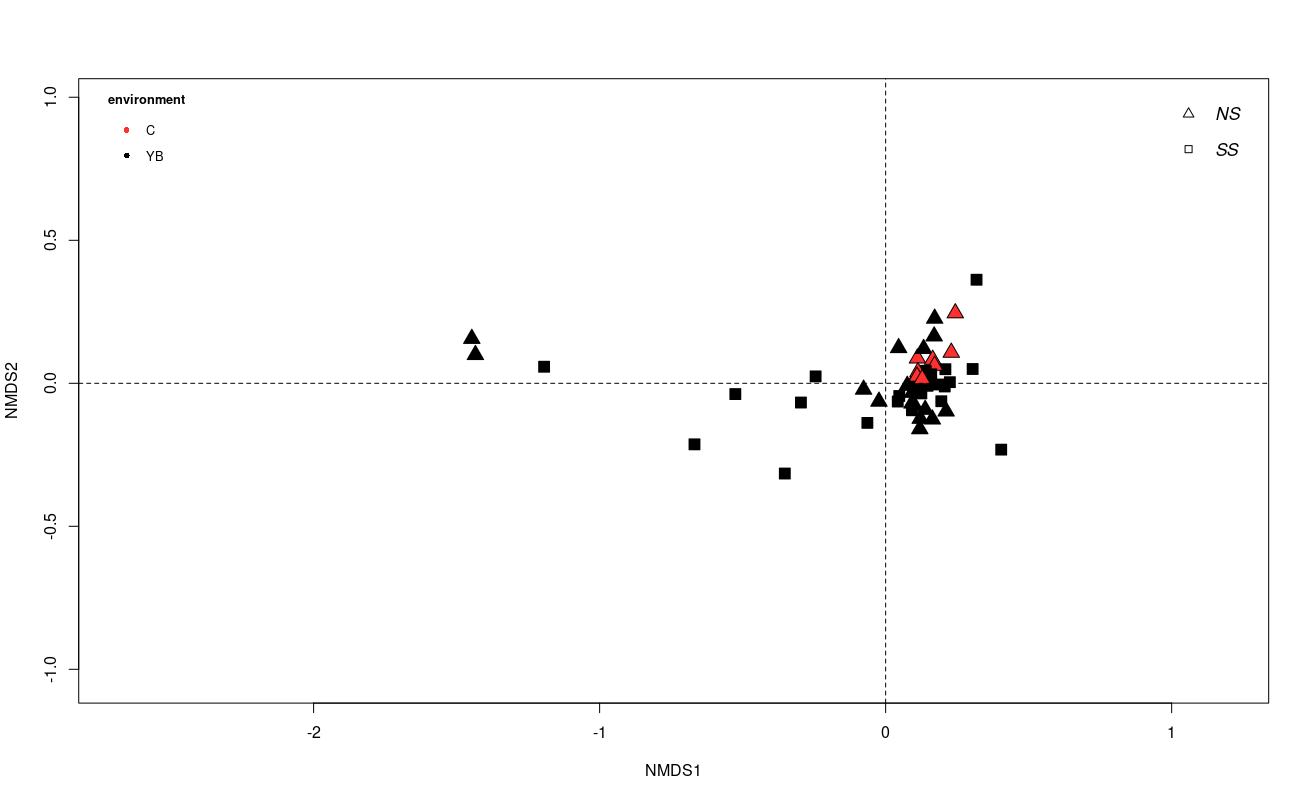
**Supplementary Figure 3.** Differences in community structure between samples as assessed using non-metric multidimensional scaling (NMDS)*.* Data from all the 52 samples were used for this analysis. Red symbols: cows (C); black symbols: young bulls (YB); triangles: EHEC O157:H7 non-shedders (NS); squares: EHEC O157:H7 shedders (S).


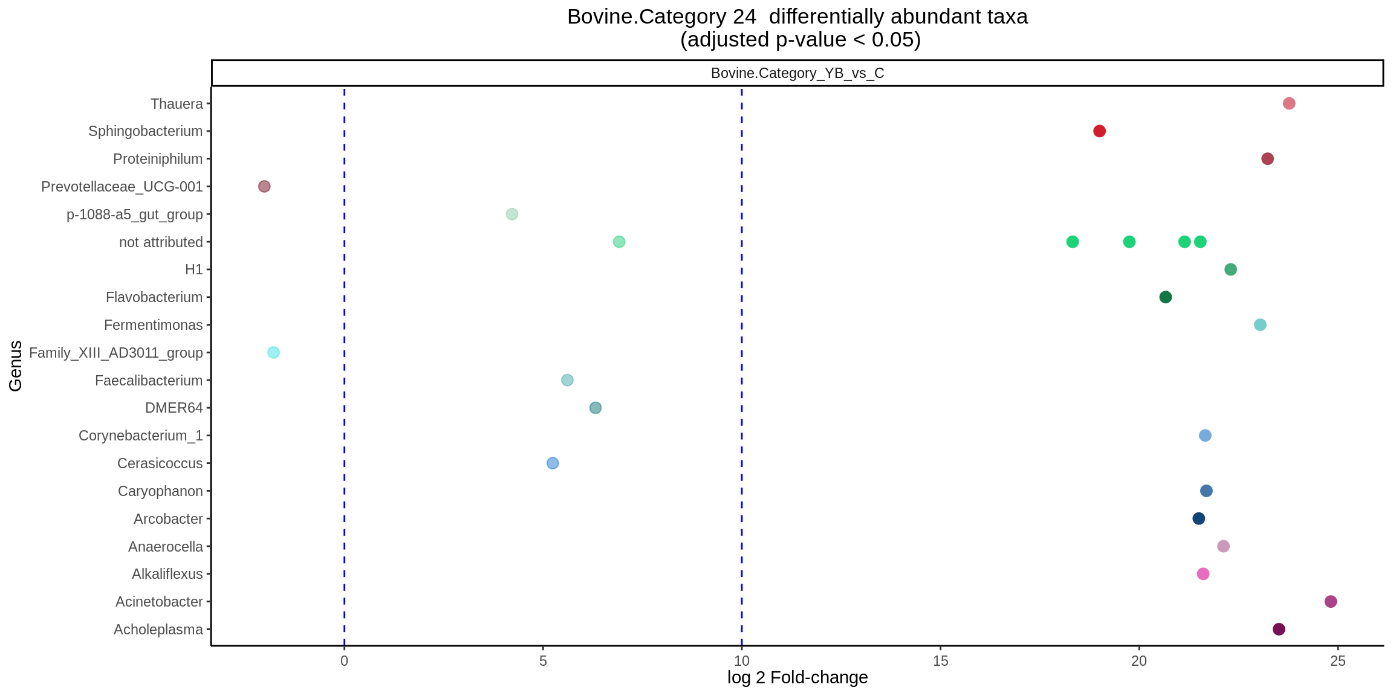


**Supplementary Figure 4.** Differential ASVs (genus level) as assessed by Log2 fold change in the fecal microbiota in cows (C) and young bulls (YB). Positive values of Log2 fold change indicate that the ASV abundance is higher in YB than in C.
